# Acoustic Behavior of Subfloor Lightweight Mortars Containing Micronized Poly (Ethylene Vinyl Acetate) (EVA)

**DOI:** 10.3390/ma9010051

**Published:** 2016-01-15

**Authors:** Luiza R. Brancher, Maria Fernanda de O. Nunes, Ana Maria C. Grisa, Daniel T. Pagnussat, Mára Zeni

**Affiliations:** 1 Laboratory of Materials Chemistry Research, University of Caxias do Sul, Rua Francisco Getúlio Vargas, 1130, Caxias do Sul RS 95070-560, Brazil; lrbrancher@ucs.br (L.R.B.); amcgrisa@ucs.br (A.M.C.G.); mzandrad@ucs.br (M.Z.); 2 Laboratory of Constructive Technology, University of Caxias do Sul, Avenida Frederico Segala, 3099, Caxias do Sul RS 95010-550, Brazil; dtpagnussat@ucs.br

**Keywords:** recycled aggregates, lightweight mortar, poly (ethylene vinyl acetate), impact noise

## Abstract

This paper aims to contribute to acoustical comfort in buildings by presenting a study about the polymer waste micronized poly (ethylene vinyl acetate) (EVA) to be used in mortars for impact sound insulation in subfloor systems. The evaluation method included physical, mechanical and morphological properties of the mortar developed with three distinct thicknesses designs (3, 5, and 7 cm) with replacement percentage of the natural aggregate by 10%, 25%, and 50% EVA. Microscopy analysis showed the surface deposition of cement on EVA, with preservation of polymer porosity. The compressive creep test estimated long-term deformation, where the 10% EVA sample with a 7 cm thick mortar showed the lowest percentage deformation of its height. The impact noise test was performed with 50% EVA samples, reaching an impact sound insulation of 23 dB when the uncovered slab was compared with the 7 cm thick subfloor mortar. Polymer waste addition decreased the mortar compressive strength, and EVA displayed characteristics of an influential material to intensify other features of the composite.

## 1. Introduction

Following the rapid growing of vertical constructions, users search, among other options, those which will bring them better acoustic comfort, since one of the main causes of discomfort in residential buildings is the lack of acoustic insulation of impact noise.

Impact noise is the noise produced from a mechanical impact and transmitted by the building structure. Usually, the impact noise is better perceived at the story below the floor where people are walking, dragging furniture, falling objects, *etc.* The usual materials employed in reducing this kind of noise and consequently increase acoustic insulation in floor systems employ principles related to the dampening of mechanical impact. However, this efficiency in dampening should not impart huge modifications to the rigidity of the floor system so as not to cause instability when a user is walking.

In this sense several studies have been developed in the search for incorporating different polymer wastes into new materials to qualify the acoustic performance in buildings. The influence of factors, such as the particle size distribution of the material incorporated into different kinds of composites, varies according to the kind of material, but as a general principle the lower the particle size, the higher is the efficiency of the material towards insulation of impact sound [1,2,3].

António *et al.* [4] studied cement-cork screeds with three varying cement proportions, three different thicknesses and their application for the reduction of impact sound in floors. In this study, as the cement content increased, the impact reduction declined, particularly when the cement-cork screed was tested as floor covering. The best results were obtained for the 3 cm thick sample. However, the samples with highest cement content give similar impact sound reduction for the three thicknesses tested.

D’Alessandro *et al.* [2] tested lightweight concrete containing polymers derived from recycled sheets of electric wires with small, 1-m² size samples. They explain that even if the developed lightweight concrete cannot be considered a traditional resilient material, as the ones to which the method is addressed, the high percentage of polymer makes the product elastic enough. The performed investigations show that it is possible to use polymeric residues in a new product showing promising acoustic and thermal performance if compared with common lightweight screeds. They conclude that the high stiffness of the material is the most important variable in impact sound reduction.

In addition to particle size, the hot or cold press process also influences, the cold processes leading to better acoustic performance since the integrity of polymer materials is maintained [5].

Studies report that the use of polymer wastes in conventional cementitious composites reduces the mechanical strength of the material as the polymer percentage is increased [6]. However, studies found a different relationship with high performance concrete. Self-Compacting Concrete (SCC) is a new class, sophisticated high performance concrete based on recycled end-of-life vehicle tires endowed with environmental, economic and performance benefits. Najim and Hall [7] indicate that the Self-Compacting Rubberized Concrete (SCRC) composite could be used for applications requiring deformable concrete with high flowability and low/medium strength (<35 MPa). The rubber aggregate was used to partially substitute fine and coarse aggregates in proportions of 5%, 10% and 15%. They concluded that SCRC has mechanical properties sufficient for structural applications, but with superior dynamic properties relative to conventional mixes.

Borzog-Haddad *et al.* [8] claim that several studies have demonstrated that structural elements made of post-consumer recycled polymers can be used in construction. Nevertheless, some precautions should be observed due to the importance of accelerated creep test methods for polymeric products. Creep is a deformation of a viscoelastic material under a constant load, there being a strong relationship between time, creep strain and applied stress. The magnitude of creep depends on the type of polymer and the manufacturing processes, which cause a large difference in the creep behavior among different polymeric products. Therefore, the creep of each polymeric product should be evaluated so that appropriate reduction factors can be applied in design.

However, this material can have non-structural applications, which do not require the high mechanical strength of constructive elements, for which the increase in polymer amount could mean higher efficiency in thermal and acoustic insulation [9,10,11].

The footwear industry of South Brazil produce every month huge amounts poly (vinyl ethylene acetate) (EVA) wastes, a thermosetting polymer which is not biodegradable and which cannot be reprocessed, which renders it a low-cost raw-material. Tutikian *et al*. [9] tested the impact sound reduction of composites made with two types of recycling processes with and without treatment to the EVA powder generated during the process. The authors found similar results for the two cases, which indicates that a strict control in the production of the composites is not required, which could represent some reduction in production costs.

In this context, this study aims at evaluating the influence of micronized EVA waste on the physical, mechanical and morphological properties of subfloor mortars in order to improve their properties of impact noise insulation in residential buildings. The impact sound insulation test was carried out with subfloor samples with 50% EVA waste in order to check the efficiency of the highest possible incorporation of polymer.

## 2. Procedure

A methodology was established to characterize the subfloor samples behavior considering the use with a concrete slab of typical Brazilian residential buildings.

EVA micronized waste was supplied by a footwear industry from Rio Grande do Sul State/Brazil. CP IV cement was supplied by Votorantim and as natural aggregate medium sand was employed, classified as small aggregate. Sand was previously dried in an oven at the temperature of 100 °C being kept in a sealed vessel until use.

### 2.1. Methods of Preparation

The preparation of the subfloor mortar was carried out in agreement with the NBR 13276 [12] Brazilian Standard. In order to determine the amounts to be employed, a proportion of 1:4 (binder: aggregate) by volume and corresponding amount of 1:6.5 by mass was established. For the subfloor mortar, the proportion by mass was prepared by replacing 0%, 10%, 25% and 50% of the natural aggregate by the EVA micronized waste having average particle size 401.45 µm and specific weight 1.11 g/cm³, according to the NBR NM 23 Brazilian Standard and unit mass 0.16 g/cm³ in accordance with the NBR NM 45 [13] Brazilian Standard.

Table 1 lists the proportions employed for each mortar sample and the water/binder ratio which was not obeyed since the polymer has a huge capacity for water absorption.

**Table 1:** Proportions employed in the preparation of samples.

Samples	Proportions (Mass)	Water/Binder Ratio
Reference Mortar	1:6.5	0.92
Mortar 10% EVA	1:0.068:5.82	0.55
Mortar 25% EVA	1:0.17:4.85	0.97
Mortar 50% EVA	1:0.34:3.23	0.65

After homogenization, cylindrical 5 cm diameter × 10 cm height and prismatic test specimens 4 cm × 4 cm × 16 cm were molded in order to perform mechanical and physical testing. Besides, 20 cm × 20 cm surface area plates were prepared, of three different thicknesses: 3 cm, 5 cm and 7 cm. Test specimens remained for 28 days under cure in a climatized room at a temperature of 23 ± 2 °C and a relative humidity of 80% ± 5%.

### 2.2. Characterization

#### 2.2.1. Water Absorption, Voids Index and Actual Specific Weight

Absorption tests after water immersion at 23 °C (±2 °C) temperature, voids index after saturation in water and actual specific weight were performed in triplicate in accordance with the NBR 9778 [14] Brazilian Standard, after 28 days under cure.

Test specimens were kept in a Quimis^®^ oven at a temperature of 60 °C (±5) °C for 72 h. After drying test specimens were kept submerged in water in a climatized room at a temperature of 23 ± 2 °C, they were weighed in a Marte^®^ scale until mass stability. The test procedure includes drying the sample at a temperature of 105 ± 5 °C for a period of 72 h. The mass of the dry sample in grams is registered; thereafter, it is immersed in water at 23 ± 2 °C and kept for 72 h in this condition. After saturation, the sample is placed in a container full of water which must be gradually brought to boiling and held for 5 h. Allow the sample to cool naturally to 23 ± 2 °C and the mass of the sample immersed in water at hydrostatic balance is registered.

#### 2.2.2. Field Emission Scanning Electronic Microscopy

For the morphological analysis of the samples the same proportion of the mortar samples of 1:4 by volume was kept. Morphological analysis of the samples was performed in a Tescan^®^ model Mira 3 field emission scanning electronic microscope aiming at evaluating the polymer interface at the cementitious matrix microstructure.

#### 2.2.3. Compressive Strength

The test for compressive strength was carried out on cylindrical test specimens in triplicate, in accordance with NBR 7215 [15] Brazilian Standard in a EMIC—PC200I hydraulic press in order to evaluate the mechanical strength of the mortars modified by the EVA micronized waste.

#### 2.2.4. Compressive Creep

For the compressive creep test Mitutoyo dial indicators of 0.01 accuracy were used to measure the subfloor creep, in accordance with the procedures of the ISO 20392 [16] Standard. Mortar plates were compressed by a 200 kg/m² steel plate until stabilization of the dial indicators. Creep was estimated by regression analysis for a 13-year period, which is the minimum useful life of a subfloor in accordance with Brazilian Standard NBR 15575 [17]. The creep of reference samples and that of 10% and 50% EVA micronized waste in replacement of natural aggregate was evaluated in order to check if the thickness reduction throughout the useful life of the floor system keeps the same dampening capacity, with porosity preservation. 

#### 2.2.5. Impact Noise

The impact noise test was performed in accordance with the ISO 140-7 Standard [18] in a two-floor prototype of dimensions 2.55 m × 4.90 m × 2.58 m. The masonry of the upper floor was build from ceramic blocks and that of the lower floor from concrete blocks, both of 19 cm thickness. Rooms are separated by a 13 cm thickness structural concrete slab and have brickwork walls covered with plaster and paint.

The noise for the tests was generated with a normalized Bruel and Kjaer type 3207 tapping machine and the measurements were carried out with a Bruel and Kjaer type 2270 sound level analyzer, which has a ½” microphone.

**Figure 1:**
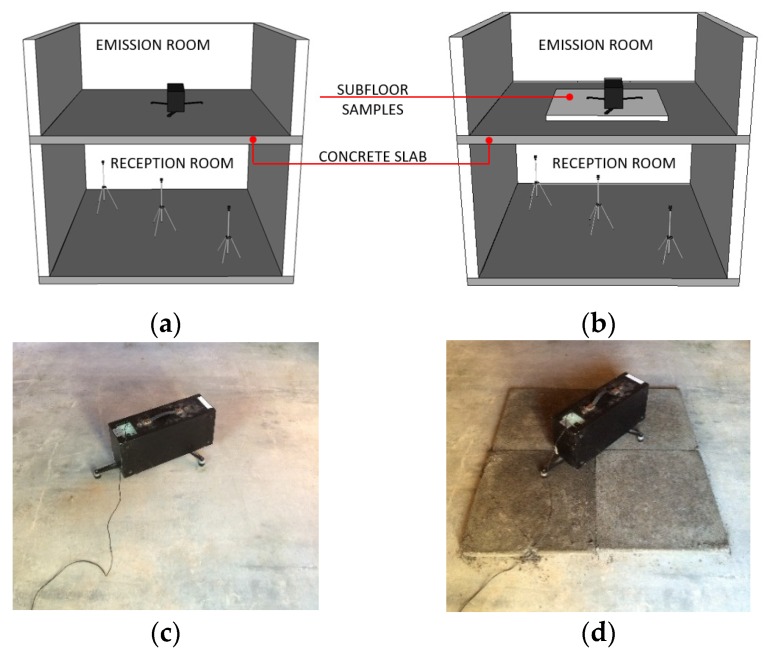
Rooms of the tests and position of the equipment: (**a**) concrete slab; (**b**) subfloor samples; (**c**) experimental setup without samples; and (**d**) with samples.

On the upper floor is placed the standard tapping machine which is in charge of emitting the mechanical impact on the subfloor, which is the origin of noise in the lower floor, which is captured by a microphone (Figure 1). Five measurements were obtained for each four positions of the instrument, at the bands of one-third octave between 100 Hz and 3.15 kHz and the result is obtained by the weighed standardized impact noise level L’_nT,w_. The test was carried out with subfloor samples with 50% EVA waste in order to check the efficiency of the highest possible incorporation of polymer.

## 3. Results and Discussion

### 3.1. Absorption of Water, Voids Index and Actual Specific Weight

By adding the polymer waste, the voids in the sample augment as a function of EVA porosity. Consequently its specific weight is reduced which contributes to reduce overburden of structure of a multi-flooring building. The increase in EVA waste content promotes increase in voids index which assures dispersion of energy at the subfloor surface layer when a percussion action is exerted on the floor, which contributes for reducing the mechanical impact to be transmitted by the slab.

Table 2 lists results for water absorption, voids index and actual specific weight, with the respective standard deviation.

**Table 2:** Results for water absorption, voids index and actual specific weight.

Samples	Water Absorption (%)	Voids Index (%)	Actual Specific Weight (kg/m³)
Reference	9.19 ± 0.18	17.95 ± 0.19	2380 ± 19
10% EVA	11.61 ± 0.26	20.81 ± 0.30	2260 ± 17
25% EVA	11.48 ± 0.76	19.49 ± 0.99	2110 ± 21
50% EVA	15.60 ± 1.62	22.45 ± 1.70	1850 ± 20

The higher water absorption of the mortar produced with EVA may be explained by the pore size and pore network formed due to the packing of grains of each sample. A discrepancy was observed for water absorption results and voids index for the 25% EVA mortar relative to the other waste percentages. This can be explained in the light of better packing of grains in this range of replacement of sand by the polymer.

### 3.2. Field Emission Scanning Electron Microscopy

**Figure 2:**
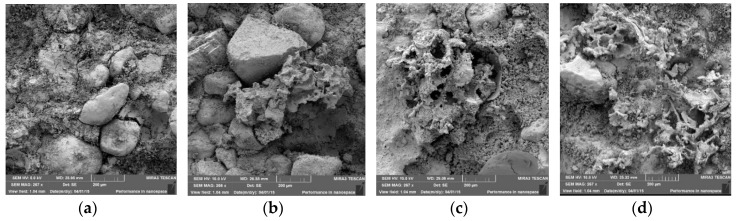
Samples Morphology: (**a**) reference mortar; (**b**) mortar 10%; (**c**) mortar 25%; and (**d**) mortar 50% EVA wastes.

The mortar morphology (Figure 2a) evidenced that there is no chemical interaction among the sands grains and the cement resulting from the aggregate property of being inert.

Mortars (Figure 2b–d) show the same morphological feature no matter the EVA content. EVA has a porous structure and cement deposits superficially on EVA, favoring the increase in mortar’s voids index.

Whenever there is no intrusion of cement particles within the EVA aggregate pores, the low rigidity of the system is kept as such, preserving dissipation of mechanical energy by the material internal dampening, with the consequent reduction in impact noise.

Energy dissipation of the mechanical impact is explained by the increased contact time derived from the dampening of kinetic energy by the elastic nature of the material. Under these circumstances the excitation spectrum keeps the same force amplitude with different distributions throughout time [19].

### 3.3. Compressive Strength

Table 3 lists data for compressive strength of the reference mortar and of mortars having 10%, 25% and 50% EVA wastes.

**Table 3:** Data for compressive strength of the mortars prepared in this study.

Samples	Compressive Strength (MPa)
Reference Mortar	5.44 ± 0.12
Mortar 10%	4.07 ± 1.45
Mortar 25%	2.07 ± 0.16
Mortar 50%	1.44 ± 0.25

Addition of EVA wastes represented reduction in compressive strength of the material. Variations from 5.44 MPa for the reference mortar down to 1.44 MPa for the mortar incorporating 50% EVA (Table 2) were observed. This behavior can be explained in the light of the high porosity of the EVA waste, which heightens the mortar voids index, reducing its mechanical strength.

Herrero *et al.* [20] studied the influence on mortars of different amounts and particle size ranges in end-of-cycle rubber. In mechanical testing it was observed that reductions in strength are more apparent for mortars of higher polymer amounts (60%) and lower particle size (0–0.6 mm). The mortar containing the highest amount of micronized polymer (50%) used in the present research with particle size of nearly 0.4 mm had the lowest mechanical strength, these results being similar to those reported by Herrero *et al.* [10].

### 3.4. Compressive Creep

**Figure 3:**
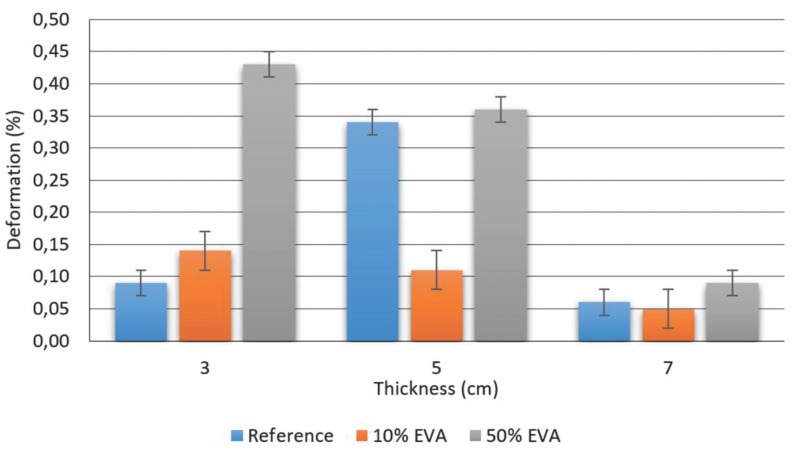
Creep results for subfloor samples.

Figure 3 illustrates the results for compressive creep, which evidences the deformation of reference, 10% and 50% EVA plates for three different thicknesses for a 13-year period.

The compressive creep deformation results were all less than 0.5%, and these values can be considered favorable to the use of the material as a non-structural element, since structural elements present satisfactory results between 20%–30% [21].

One can also compare the results with the compressive creep of resilient materials commonly used to reduce impact noise. Gnip *et al.* [22] analyzed the deformation under constant compression of mineral wool samples with seven different thicknesses and densities. Deformation was lower in samples with greater thickness, with 0.3% for 80 and 150 mm. For the thinner samples of 30 and 40 mm, the deformation was between 1% and 1.3%.

The ability of aggregates to restrain movement of a cement paste depends upon the extensibility of the paste, the degree of cracking of the paste, the compressibility of the aggregate and changes in the aggregate moisture content. Generally, concretes that have aggregates that are hard, dense and have low absorption and high modulus of elasticity are desirable when concrete with low creep is needed [23].

The lower height creep percent mortar was the 7 cm thickness, 10% EVA sample. In view of the evaluation of mortars in subfloor systems, lower creep assures the integrity of the plate throughout time and reduced diminishing of voids index, which collaborates with mechanical impact dampening during its use [19].

### 3.5. Impact Noise

Analysis of the acoustic test results were performed by comparing the noise level curve (L’_nT_) of the concrete slab as well as of each subfloor samples tested. The performance rating was obtained in L’_nT,w_.

The impact noise tests were performed with 50% EVA waste only, in order to assess the viability of the material with highest possible residue content. Furthermore, the procedure using larger samples and thorough testing with other proportions would generate more waste.

Table 4 lists results for impact noise level for samples of 50% EVA waste.

**Table 4:** Results for impact noise level for samples of 50% EVA waste.

Sample	Sample Thickness (cm)	Impact Noise Level L’_nT,w_ (dB)
50% EVA 3 cm	3	51
50% EVA 5 cm	5	58
50% EVA 7 cm	7	56
Concrete slab	13	79

Taking into consideration that the highest L’_nT,w_ level accepted by Brazilian standards is 80 dB, all the samples tested complied with such requirement. It was observed that the 3 cm thickness sample exhibited impact noise propagation lower than those of the other, thicker samples. It is well-known that as the slab thickness increases, the propagation of the impact noise of a concrete plate is reduced [3]. Therefore, a better acoustic performance is expected for the thicker plates. On receiving the mechanical impact, the subfloor degradation occurs as a result of its limited thickness, as seen in the 3 cm thickness sample which was not able to keep its integrity after the impact. Hence, the particles of the lower portion of the plate detached and dampened the mechanical impact, with consequent reduction in subfloor impact noise.

**Figure 4:**
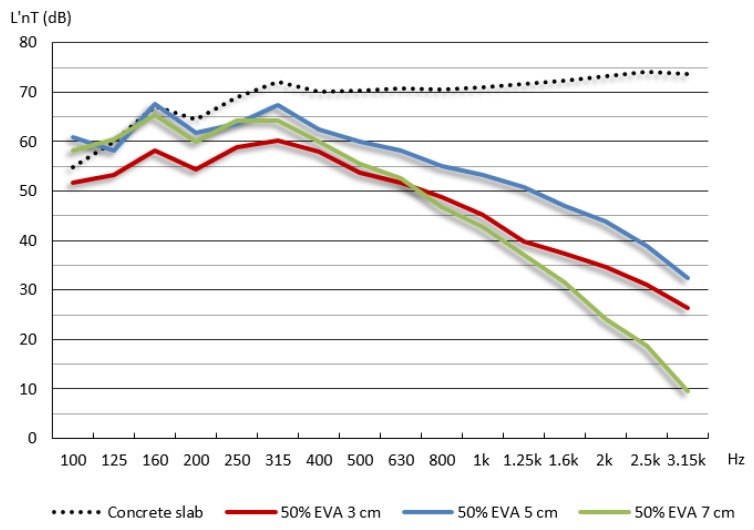
Impact noise level one-third octave band frequency for samples of 50% EVA waste.

The results of the impact noise levels can be grouped into three different zones, determined by frequency ranges up 400 Hz, between 400 Hz and 1.6 kHz and above 1.6 kHz (Figure 4). The results at low frequencies, up 400 Hz corresponded to bass sounds measured in the reception room. In this situation, the 3 cm thickness sample presented higher sound reduction comparing to concrete slab. For 160 Hz frequency range the samples with 5 and 7 cm thickness showed sound levels as high as the ones measured with the concrete slab. This could be due to the self-resonance-frequency effect, and it occurs at low frequencies and is directly proportional to the superficial density of the solid material [24]. Thus, the higher the superficial density of a specific material the higher the level of acoustic pressure.

Within middle frequency interval, from 400 Hz and 1.6 kHz, the levels of acoustic pressure reduced when the frequency was higher, *i.e*., the higher the frequency band, the more acute is the sound. Moreover, the higher the frequency, the lower the measured sound pressure level measured in the reception room with EVA waste subfloor samples.

In the zone of higher frequencies, from 1.6 kHz, there was an increase of the sound insulation, especially for reduced sound level with 7 cm thickness subfloor sample. In this sample, a mortar detachment of particles was observed. This effect was caused by loose particles of reduced size which can, under certain circumstances even increase the efficiency of a floor system. However, these particles can lead to moves that jeopardize the integrity of the whole floor system [25].

It should be emphasized that this effect can be suppressed with a final coating material, which despite being considered a complementary solution, in this case can significantly contribute to the attenuation of impact sound transmission. This could be attributed to the fact that the time of impact percussion in final covering floors is smaller than the impact time of a percussion carried out in the uncovered floor [19].

The effect of substitution of natural aggregates for lightweight aggregate to reduce the impact noise has also been observed in other studies. Branco and Godinho [3] found significant reductions in noise levels of lightweight mortars with cork, polystyrene and clay, mainly in the frequency bands above 400 Hz, when compared with conventional mortar subfloor. In another study, Olmeda *et al.* [26] incorporated petroleum coke to produce lightweight mortars and also identified sound levels reductions with increased frequency bands. The most significant differences were of 1.6 kHz.

## 4. Conclusions

Samples having incorporated the EVA polymer waste had favorable features for impact noise insulation. Addition of the waste favored the rise in voids index which contributes to reduce the subfloor actual specific weight rendering lighter the system, with benefits not only to the building general structure in terms of reduction of structural overcharge, but also to impact noise attenuation transmitted by the slab at each floor. In view of the non-utilization of the cementitious composite in structural buildings, reduction of compressive strength resulting from waste incorporation is not a determining factor for the suggested use according to the present study. The long term lower subfloor creep, as estimated by compressive creep tests assures the system integrity, allowing the subfloor to keep the property of impact noise dampening, providing better acoustic comfort to users of residential buildings.
